# MiR-181b sensitizes glioma cells to teniposide by targeting MDM2

**DOI:** 10.1186/1471-2407-14-611

**Published:** 2014-08-25

**Authors:** Yan-chang Sun, Jing Wang, Cheng-cheng Guo, Ke Sai, Jian Wang, Fu-rong Chen, Qun-ying Yang, Yin-sheng Chen, Jie Wang, Tony Shing-shun To, Zong-ping Zhang, Yong-gao Mu, Zhong-ping Chen

**Affiliations:** Department of Neurosurgery/Neuro-oncology, Sun Yat-sen University Cancer Center, State Key Laboratory of Oncology in South China, Guangzhou, Guangdong China; Department of Neurosurgery, Guangdong Medical College, Zhanjiang, Guangdong China; Department of Health Technology and Informatics, The Hong Kong Polytechnic University, Hung Hom, Hong Kong; Department of Neurosurgery, Hainan Provincial Nongken Hospital, 48 Bashuitang Road, Longhua Distric, Haikou, Hainan 570311 China

**Keywords:** miR-181b, Teniposide, Glioma, Mouse double minute 2 homolog (MDM2)

## Abstract

**Background:**

Although the incidence of glioma is relatively low, it is the most malignant tumor of the central nervous system. The prognosis of high-grade glioma patient is very poor due to the difficulties in complete resection and resistance to radio-/chemotherapy. Therefore, it is worth investigating the molecular mechanisms involved in glioma drug resistance. MicroRNAs have been found to play important roles in tumor progression and drug resistance. Our previous work showed that miR-181b is involved in the regulation of temozolomide resistance. In the current study, we investigated whether miR-181b also plays a role in antagonizing the effect of teniposide.

**Methods:**

MiR-181b expression was measured in 90 glioma patient tissues and its relationship to prognosis of these patients was analyzed. Cell sensitivity to teniposide was tested in 48 primary cultured glioma samples. Then miR-181b stably overexpressed U87 cells were generated. The candidate genes of miR-181b from our previous study were reanalyzed, and the interaction between miR-181b and target gene MDM2 was confirmed by dual luciferase assay. Cell sensitivity to teniposide was detected on miR-181b over expressed and MDM2 down regulated cells.

**Results:**

Our data confirmed the low expression levels of miR-181b in high-grade glioma tissues, which is related to teniposide resistance in primary cultured glioma cells. Overexpression of miR-181b increased glioma cell sensitivity to teniposide. Through target gene prediction, we found that MDM2 is a candidate target of miR-181b. MDM2 knockdown mimicked the sensitization effect of miR-181b. Further study revealed that miR-181b binds to the 3’-UTR region of MDM2 leading to the decrease in MDM2 levels and subsequent increase in teniposide sensitivity. Partial restoration of MDM2 attenuated the sensitivity enhancement by miR-181b.

**Conclusions:**

MiR-181b is an important positive regulator on glioma cell sensitivity to teniposide. It confers glioma cell sensitivity to teniposide through binding to the 3’-UTR region of MDM2 leading to its reduced expression. Our findings not only reveal the novel mechanism involved in teniposide resistance, but also shed light on the optimization of glioma treatment in the future.

## Background

Gliomas are the most common primary brain tumors. There are around 10,000 new cases of high-grade gliomas each year in the United States [1]. Malignant gliomas are currently treated by surgery followed by radiotherapy and chemotherapy [2]. Although cancer survivors are estimated to be over 13.7 million in the United States [3], the five-year survival rate of the most malignant glioma, glioblastoma multiforme(GBM), is only 9.8% at the best due to difficulties in complete resection and the low sensitivity to radio-/chemo- therapeutic agents [4–6]. Therefore, finding ways to sensitize glioma cells to both radiotherapy and chemotherapy is of great importance.

MicroRNAs (miRNAs), a class of small noncoding RNA of 20–22 nucleotides in length, are processed from larger pre-miRNAs by the RNase III enzyme Dicer (DICER1) into miRNA duplexes. One strand of the duplex will then associate with the RNA-induced silencing complex (RISC), and the other will be degraded by nucleases. The RNA-RISC complex will specifically bind to the mRNA target, leading to target degradation and subsequent translational silencing. MiRNA regulates the expression of downstream target genes and involves in tumorigenesis as well as tumor sensitivity to treatment [7]. There is evidence showing that miRNA could also function as both tumor suppressors and oncogenes. MiR-181b has been shown to be a tumor suppressor. It functions as an inhibitor in tumor growth, colony formation and invasion [8–10]. MiR-181b is downregulated in tumor tissues and glioma cell lines. It is much lower in cancer stem cells enriched from the glioma cell line U87. The ectopic expression of miR-181b suppressed glioma cell colony formation, proliferation and reduced their resistance to temozolomide [11].

Teniposide (Vumon, VM-26), a semi-synthetic derivative of podophyllotoxin resin, is a cell cycle specific cytotoxic drug that inhibits the activity of DNA topoisomerase-II and stabilizes the DNA-Topo II complex in DNA replication, thus damaging DNA and inducing cellular apoptosis. Teniposide has been used successfully in treating several neoplastic disorders, such as lung and ovarian cancers as well as squamous cell carcinoma [12–14]. Teniposide has gradually become the effective chemotherapeutic drug for intracranial malignant tumors due to its low cytotoxicity, high lipid solubility and small molecular weight, all of which facilitates its passage through the blood-brain barrier [15, 16]. Although the initial response of teniposide is remarkable, tumor resistance develops rapidly after prolonged administration [17]. Therefore, it is of therapeutic significance to determine how to promote drug sensitivity, and therefore reduce application dose. In this study, we confirmed the expression of miR-181b decreases with increasing grade in gliomas. More importantly, we demonstrated that miR-181b promotes the sensitivity of glioma cells to teniposide through direct modulation of the level of MDM2.

## Methods

### Patients and cell lines

Patient tumor samples were collected, with written informed consent, from the Department of Neurosurgery/neuro-oncology, Sun Yat-sen University Cancer Center (SYSUCC) between 2001 and 2008. Primary tumor cultures were derived from the tissue samples using the method described by Brassesco et al. [18]. Human GBM cell line U87 (maintained in SYSUCC) were obtained from American Type Culture Collection. All cells were maintained in DMEM (Gibco) supplemented with 10% fetal bovine serum (FBS, Hyclone) and 1% penicillin–streptomycin (Gibco) at 37°C in a humidified incubator with 5% CO_2_. The Ethics Review Board of SYSUCC approved this study.

### Primary cell culture

Fresh tumor samples (verified from frozen sections), aseptically collected in the operating room, were minced with scissors in a petri dish. The tumor pieces were then disaggregated for 4 h at 37°C in 0.5% type IV collagenase (Sigma-Aldrich, St Louis, MO, USA) in F10 medium (Life Technologies, Carlsbad, CA, US). Then, the cells were pelleted and resuspended in the medium with 15% FBS. Cells were used for experiments after being cultured for 1 week.

### RNA extraction and real-time PCR analysis

RNAs from 90 glioma samples and 4 normal brain tissues (normal adjacent tissues) were extracted with Trizol reagent (Invitrogen, USA) accordingly. RNA concentration and purity were measured using the NanoDrop ND-1000 Spectrophotometer (Thermo Fisher). One μg of total RNA was used for cDNA synthesis by the First Strand cDNA Synthesis Kit (Thermo-scientific, PA, USA). Real-time PCR reaction was performed as follows: 94°C 4 min for hot start, and then 94°C for 30 sec, 50°C for 30 sec, 72°C for 40 sec, for 40 thermal cyclesusing SYBR Premix Ex Taq II kit (TaKaRa, Dalian, China) on an ABI 7900HT instrument (ABI, NY, USA). The primers for miR-181b and U6 endogenous controls were purchased from RiboBio (Guangzhou, China). Primers for the MDM2 and GAPDH controls were from Invitrogen. All reactions were performed in triplicates. Relative gene expression was calculated using the 2-^ΔΔ^CT method.

### Chemosensitivity assay

Cells (primary cultured cells and U87 cell line) were seeded onto 96-well plates at 2,000 cells per well in triplicates. The next day, cells were treated with teniposide at a concentration gradient of 50, 25, 12.5, 6.25, 3.125, 1.5625, 0.78125, 0.39, 0.19, and 0 μg/ml. Cell viability was measured after 72 h using the Cell Counting Kit 8(CCK8, Dojindo, Japan) according to the manufacturer’s instructions. IC50 for teniposide was calculated from the dose-dependent curve.

### miR-181b lentiviral transfection

The effect of miR-181b on cell sensitivity to teniposide was evaluated by constructing stable miR-181b-expressing glioma cells. The lenti-miR-181b and the corresponding empty vector were purchased from GenePharma (Shanghai, China). U87 was selected due to its low basal level of miR-181b. After being incubated with the virus for 72 h, cells were selected by medium containing 2 μg/ml puromycin for about 1 week.

### miR-181b target gene prediction

The candidate targets of miR-181b were predicted by the following applications: Target scan (http://www.targetscan.org), PicTar (http://www.pictar.org), and database (http://www.mirbase.org).

### Dual luciferase reporter assay

The plasmids psiCHECK-wtMDM2 and psiCHECK-mutMDM23’UTRs (carrying a mutational miR-181b binding site) were purchased from Land (Guangzhou, China). In brief, the full length MDM2 and empty psiCHECK vector were digested by same restriction endonuclease, followed with ligation, transformation, and then confirmed by both digestion and sequencing. The mutant MDM2 sequence was generated by mutagenesis PCR reaction from psiCHECK-wtMDM2. Then similar procedures (endonuclease digestion, ligation, transformation and confirmation) were performed to get psiCHECK-mutMDM2. 293 T cells were seeded onto 24-well plates. After overnight incubation, they were co-transfected with 50 nM miR-181b mimic or non-relevant control (NC), together with 0.5 μg reporter vector containing either wtMDM2 3’UTR or mutMDM23’UTR. Cells were harvested 48 h after transfection. The renilla and firefly luciferase activity were determined byDual Luciferase Assay Kit (Promega) following manufacturer’s instructions. Data were presented as mean ratio of the renilla/firefly luciferase activity obtained from at least three independent experiments [19].

### Western blot analysis

Cells were washed twice with ice-cold PBS and lysed in RIPA buffer containing 1 mM PMSF (Beyotime, Japan) on ice [20]. Lysates were centrifuged at 12,000 × g for 10 min at 4°C, and supernatants were collected. Equal amounts of proteins (20 μg) were fractionated by SDS-PAGEand transferred onto nitrocellulose membranes (Millipore, MA, USA). After being blocked in 5% nonfat milk at room temperature for 1 h, membranes were probed with primary antibodies at 4°C overnight. The next day, secondary antibodies were incubated for 1 h at room temperature and visualized using enhanced chemiluminescence (Millipore, MA, USA). The following antibodies were used: mouse anti-β-actin (1:1000, Santa Cruz, Dallas, TX, USA), rabbit anti-MDM2 (1:5000, Abcam, Cambridge, MA, USA), anti-phospho-MDM2 (1:1000, Cell signaling technologies, Boston, MA, USA).

### Statistical analysis

The correlation between miR-181b expression and patient survival was analyzed by the Kaplan-Meier survival curve method. The correlation between miR-181b and glioma sensitivity to teniposide was determined by Spearman’s correlation coefficient. Differences between groups were calculated using paired *t*-test or one-way ANOVA. p < 0.05 was considered statistically significant. All statistical analyses were performed using SPSS, version 16.0.

## Results

### Expression of miR-181b is related to poor prognosis of glioma patients

**Figure 1:**
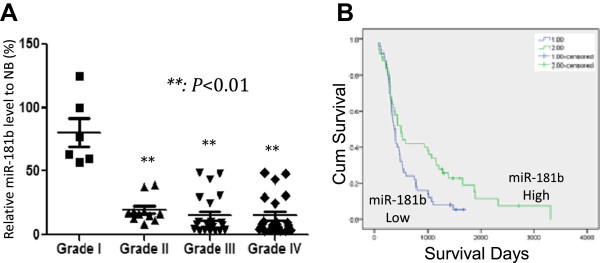
**MiR-181b expression is negatively related to glioma grade. A**: The expression of miR-181b is low in high-grade (Grade II to Grade IV) while high in low-grade (Grade I) gliomas (**: p < 0.01). **B**: The survival rate of 90 glioma patients showed that high level of miR-181b is a good prognostic marker for longer survival (p < 0.05).

A total of 90 glioma samples (WHO grade I to grade IV) were included in this study. Their cDNA were subjected to real-time PCR analysis on the expression level of miR-181b. We found that miR-181blevel was high in normal brain (3.69 ± 0.477) and low-grade glioma tissues (2.56 ± 0.354 in grade I gliomas) but very low in the high grade ones (0.067 in grade IV gliomas) (Figure 1A, p < 0.01). The Kaplan-Meier analysis showed that the median survival of patients with high expression levels of miR-181b was 493 ± 60 days, but only 370 ± 37 days in the low expression group (Figure 1B, p < 0.05). These data were consistent with previous reports emphasizing the important role of miR-181b in glioma progression.

### MiR-181b level correlates with glioma cell sensitivity to teniposide

**Figure 2:**
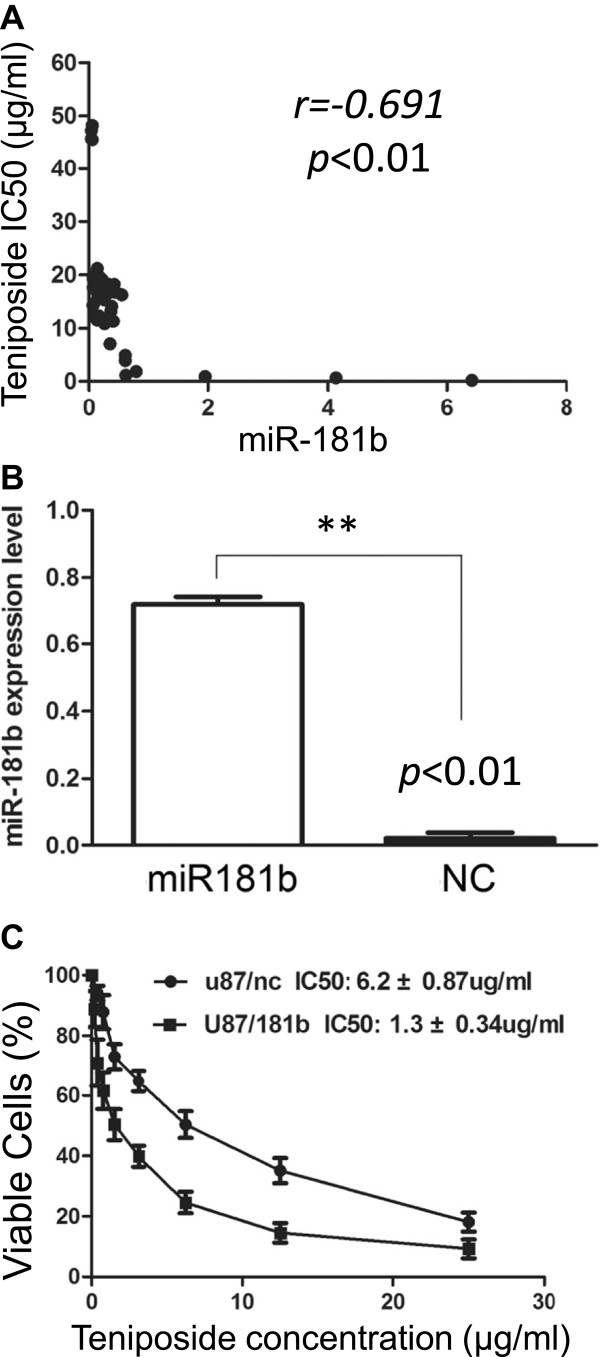
**MiR-181b level is positively related to glioma cell sensitivity to teniposide. A**: Cells expressing low levels of miR-181b had high IC50, while cells with high miR-181b had low IC50 (r = -0.691, p < 0.01). **B**: MiR-181b was successfully overexpressed in U87 cells compared with vector control (*p < 0.01). **C**: The IC50 of miR-181b overexpressed U87 cells was significantly lower than control cells (1.3 ± 0.34 μg/ml versus 6.2 ± 0.87 μg/ml).

Primary cultured glioma cells from 48 patients were treated with teniposide. The data showed that cells were highly sensitive to teniposide when expressing high levels of miR-181b, while cells showed resistance to teniposide when miR-181b level was low (Figure 2A). Expression of miR-181b in lentivirus-infected U87 cells was determined by real-time PCR (Figure 2B). The modified U87 cells were subjected to teniposide treatment and cell viability evaluated. MiR-181b overexpressed cells were more sensitive to teniposide and the number of viable cells was much lower than control cells. The IC50 was 1.3 ± 0.34 μg/ml versus 6.2 ± 0.87 μg/ml respectively (Figure 2C). These data demonstrated that miR-181b could promote the sensitivity of glioma cells to teniposide.

### MDM2 is a target of miR-181b

**Figure 3:**
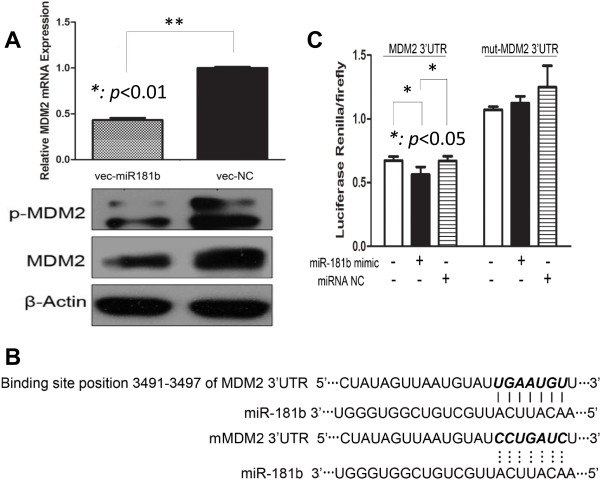
**MDM2 is a downstream target of miR-181b. A**: The mRNA level of MDM2 in miR-181b overexpressed U87 cells was reduced. The protein levels of total MDM2 and phospho-MDM2 were all reduced in miR-181b overexpressed cells compared with vector control (p < 0.01). **B**. MiR-181b could bind to the 3’-UTR region of MDM2 (3491 to 3497), while the binding was interrupted with mutated MDM2. **C**: Dural luciferase reporter assay confirmed that miR-181b mimic binds to the 3’-UTR region of wild type MDM2 but not to the mutated form (p < 0.05).

We were wondering how miR-181b mediating the sensitization of glioma cells to teniposide. From well-known databases for miRNA targets prediction, we found that mouse double minute 2 (MDM2), an important negative regulator of the p53 tumor suppressor, is one of the candidate targets of miR-181b. Microarray from our group also showed that MDM2 is one of the 85 downregulated genes in glioma cells with stable overexpression of miR-181b [19]. Consistently, we found both mRNA and protein levels of MDM2 were dramatically reduced after miR-181b overexpression, and phospho-MDM2 was decreased consequently (Figure 3A). To further confirm the interaction between MDM2 and miR-181b, we constructed the wild type and mutant MDM2 for dual luciferase reporter assay (Figure 3B). As expected, miR-181b bound to wild type of MDM2 but not to the mutant type (Figure 3C).

### Downregulation of MDM2 sensitizes glioma cells to teniposide

**Figure 4:**
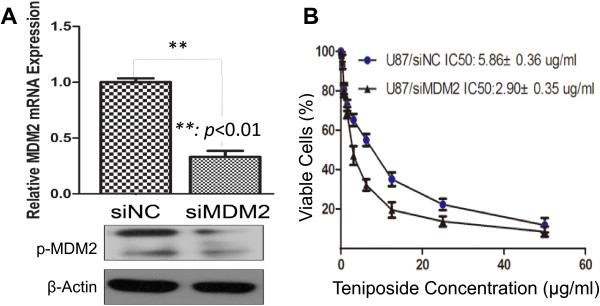
**Downregulation of MDM2 promotes cell sensitivity to teniposide. A**: The mRNA (p < 0.01) and phosphate protein level of MDM2 were all reduced after transient transfection of siRNAs in U87 cells. **B**: The IC50 of U87 cells to teniposide dropped from 5.86 ± 0.36 μg/ml to 2.90 ± 0.35 μg/ml upon the knockdown of MDM2.

Next we sought to find out whether downregulation of MDM2 could mimic the promotion of cell sensitivity to teniposide by miR-181b. We knocked down *MDM2* by siRNA and successfully reduced the mRNA level of MDM2 and protein level of phospho-MDM2 significantly (Figure 4A). After being treated with teniposide, cells with low MDM2 showed decreased viability compared with control cells, and theIC50 decreased from 5.86 ± 0.36 μg/ml to 2.90 ± 0.35 μg/ml upon MDM2 suppression (Figure 4B). These data suggested that downregulation of MDM2 could fully mimic the effect of miR-181b in increasing glioma cell sensitivity to teniposide.

### MiR-181b promotes glioma cell sensitivity to teniposide through MDM2

**Figure 5:**
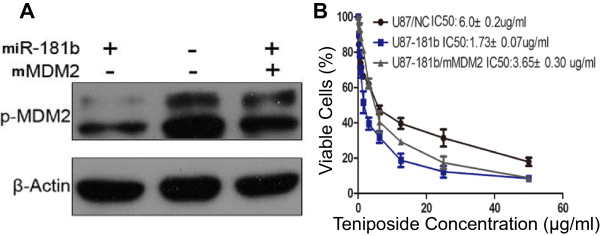
**Upregulation of miR-181b enhances cell sensitivity to teniposide through mediation of MDM2. A**: Successful overexpression of miR-181b and mutated MDM2 was confirmed by Western blot analysis. **B**: Transfection of mutated MDM2 competed the binding between miR-181b and wild type of MDM2, which reversed the teniposide sensitivity enhancement by miR-181b.

To determine if miR-181b-enhanced glioma cell sensitivity to teniposide was directly mediated by MDM2, we transfected glioma cells with miR-181b alone or together with mutant MDM2. Comparing with the vector control (Figure 5A, lane 2), the phospho-MDM2 level was reduced when cells were transfected with miR-181b alone (Figure 5A, lane 1). It was partially restored when co-transfected with mutant MDM2 (Figure 5A, lane 3). As expected, miR-181b transfection alone decreased the glioma cell sensitivity to tenopiside, IC50 of 1.73 ± 0.07 μg/ml versus 6.0 ± 0.2 μg/ml in the control cells (Figure 5B). Partial restoration of MDM2, thus the phospho-MDM2 levels, through the co-transfection of mutant MDM2 led to an increase in IC50 levels (3.65 ± 0.3 μg/ml). These results indicated that the level of phospho-MDM2 is responsible for glioma cell sensitivity to teniposide. Thus, we demonstrated that miR-181b enhances glioma cell sensitivity to teniposide through targeting E3-ligase MDM2.

## Discussion

MiR-181b has already been investigated in a number of cancer types. It is overexpressed in gastric cancer tissues and its expression in culture gastric cancer cells promotes cell proliferation, migration and invasion; whereas targeting miR-181b could lead to increased apoptosis [21]. MiR-181b also involves in hepatocarcinogenesis through promoting growth, clonogenic survival, migration and invasion of hepatocellular carcinoma cells [22]. In colorectal cancer, miR-181b is also overexpressed in tumor tissues compared with normal colorectal samples [23]. Although overexpression of miR-181b has been reported in several malignant cancers, its level in glioma is unexpectedly low. Zhi et al. found that low level of miR-181b expression in glioma tissues, through screening the miRNA expression profile of 84 astrocytomas and 20 normal adjacent tissues, is associated with poor patient survival [24]. They further validated their findings in another sample set with 40-paired astrocytoma and normal adjacent tissues. From immunohistochemistry and *in situ* hybridization assays, Tao et al. found that miR-181b is expressed at a lower level in high-grade glioma, compared with low-grade glioma [25]. The data from this study added support to previous findings where miR-181b expression is inversely related to the grading of glioma, and the expression level is a good indication of prognosis.

MiR-181b has also been reported to play roles in drug resistance in various cancers. It was found to be associated with the strong response to *S-1* (the fourth generation product of 5-fluoroufacil) when expressed low, which provided evidence for the clinical application of miR-181b as an indicator for chemoresponse of S-1 [26]. Enforced overexpression of miR-181b sensitized the human multidrug resistant cell lines SGC7901/vincristine (gastric cancer) and A549/cisplatin (lung cancer) to vincristine and cisplatin respectively [27]. Our study demonstrated that low levels of miR-181b are related to the high resistance to teniposide in primary glioma cells. By contrast, enforced expression of miR-181b in the glioma cell line U87 promoted teniposide sensitivity.

MiR-181b expression level was found to be significantly lower in poor prognostic chronic lymphocytic leukemia (CLL) patients [28]. Further study showed that transfection of miR-181b mimics could induce apoptosis in CLL cells with wild type p53, but had no effect in p53 attenuated cells [29]. Thus, the role of miR-181b in apoptosis and drug response is related to the p53 status. The link between miR-181b and p53 is now provided by the data in our study. Through overexpression of miR-181b, siRNA knockdown of MDM2 and partial restoration of functional MDM2, we have shown that the effect of miR-181b is via the phosphorylation of MDM2. The latter is a critical E3-ligase to suppress p53 protein level through ubiquitination-mediated degradation [30]. The direct effect of miR-181b on MDM2 could further lead to the reversion of p53 degradation mediated by MDM2. Taken together, miR-181b turns out not only to be a very important microRNA in carcinogenesis, but also plays a critical role in regulating drug sensitivity. Our finding may shed light on how to reduce the side effect and enhance the cell sensitivity of teniposide in treating glioma patients.

## Conclusions

MiR-181b is expressed at low levels in high-grade gliomas and can be used as a prognostic marker for glioma patients. Low level of miR-181b is related to teniposide resistance mediated through MDM2.
